# Sex Differences in Prevalence of and Risk Factors for Carotid Plaque among Adults: A Population-based Cross-Sectional Study in Rural China

**DOI:** 10.1038/srep38618

**Published:** 2016-12-06

**Authors:** Wei Zhao, Yanan Wu, Min Shi, Lingling Bai, Jun Tu, Zaiyu Guo, Rongcai Jiang, Jianning Zhang, Xianjia Ning, Jinghua Wang

**Affiliations:** 1 Department of Neurology, Tianjin TEDA Hospital, Tianjin, 300457, China; 2 Department of Neurology, Tianjin Medical University General Hospital, Tianjin, 300052, China; 3 Department of Epidemiology, Tianjin Neurological Institute, Tianjin 300052, China; 4 Department of Neurosurgery, Tianjin Medical University General Hospital, Tianjin 300052, China; 5 Department of Neurotrauma, Tianjin Neurological Institute, Tianjin 300052, China; 6 Center of Clinical Epidemiology, Tianjin Medical University General Hospital, Tianjin, 300052, China

## Abstract

Although the associations between carotid plaque and cardiovascular disease risk factors have been identified in previous studies, there is limited information on sex-related differences in factors associated with the development of carotid plaque. We aimed to determine sex differences in the prevalence of carotid plaque and associated risk factors in rural China. A total of 3,789 subjects aged ≥45 years without history of stroke or cardiovascular disease were recruited to the study. B-mode ultrasonography was performed to determine the presence of carotid plaque. The mean age of male subjects was greater than that of female subjects. In addition, there was a higher prevalence of carotid plaque in men than in women (50.1% vs. 35.5%; P < 0.001) irrespective of age group, education level, and presence of risk factors. Older age, hypertension, diabetes mellitus, and high concentrations of low-density lipoprotein cholesterol were pronouncedly associated with the risk of carotid plaque in both men and women. These findings suggest that it is vital for physicians to be aware that conventional risk factors and other related factors are of equal importance among rural residents in China; patients should thus be treated accordingly so that reduce the burden of stroke and cardiovascular disease.

Cardiovascular disease (CVD), including ischemic heart disease and stroke, is a leading cause of death in both developed and developing countries worldwide^1^. The aging and growth of populations has led to an increase in the total number of cardiovascular deaths, accounting for almost one-third of all deaths globally in 2013. Ischemic heart disease, ischemic stroke, and hemorrhagic stroke continue to cause the most cardiovascular-related and circulatory-related deaths in almost all countries^2^. Death due to CVD accounted for more than 40% of total deaths (44.6% in rural areas and 42.5% in urban areas) in China^3^. The economic burden of CVD in China between 2005 and 2015 was estimated to be approximately 550 billion USD^4^.

Carotid artery atherosclerosis is a strong predictor of ischemic stroke events as a result of both luminal stenosis and plaque rupture^5^,^6^. Carotid atherosclerosis has also been shown to be associated with an increased risk of CVD^7^,^8^.

Atherosclerotic plaques are prone to rupture because of their intrinsic composition, as they may have intra-plaque hemorrhaging and large lipid cores; consequently, atherosclerotic plaques are associated with subsequent thromboembolic ischemic events^9^,^10^,^11^. Several previous studies have indicated that carotid plaque and carotid intima-media thickening are risk factors for future CVD and cerebrovascular diseases^12^,^13^,^14^,^15^,^16^.

Sex-related differences are becoming increasingly recognized as potentially important factors in atherosclerosis^17^. Moreover, plaques in men seemed to have higher rates of erosion, ulceration, and subsequent healing^18^. Although associations between carotid plaque and CVD risk factors have been identified in previous studies^19^,^20^,^21^,^22^, studies on sex-related differences in risk factors associated with the development of carotid plaque are rare. Therefore, we aimed to assess sex-related differences in the prevalence of and risk factors for carotid plaque among adults aged 45 years and older in rural China.

## Results

### Selection of the study population and response rate

A total of 5,380 residents aged 45 years or older were qualified to participate in this study. Of these, 4,012 residents agreed to participate, resulting in a response rate of 75%. Finally, 3,789 residents participated in this study, after the exclusion of 223 residents with a previous history of CVD or stroke.

### Sex differences in demographic characteristics and physical examination findings

Of the 3,789 participants in this study, 1,560 (41.2%) were men and 2,229 (58.8%) were women. Compared to women, there were more men aged 65–74 years (21.7% vs. 17.3%; P = 0.001), aged ≥75 years (10.3% vs. 7.0%; P < 0.001), and with >6 years of education (46.4% vs. 31.9%; P < 0.001). Compared to men, there were more women aged 45–54 years (36.2% vs. 27.6%; P < 0.001); moreover, women had a higher illiteracy rate (23.4% vs. 8.8%; P < 0.001). Women had greater body mass indexes and total cholesterol (TC), triglycerides (TG), high-density lipoprotein cholesterol (HDL-C), and low-density lipoprotein cholesterol (LDL-C) concentrations than men (all P < 0.001), but systolic and diastolic blood pressure levels were greater in men than in women (P = 0.002 and P < 0.001, respectively; Table 1).

### Sex differences in the prevalence of risk factors

As shown in Table 2, there was a higher prevalence of hypertension, current smoking, alcohol consumption, and low HDL-C in men, but a higher prevalence of obesity, passive smoking, high TC, high TG, and high LDL-C in women (all P < 0.001). The prevalence of diabetes mellitus was similar between men and women (P = 0.719).

### Sex differences in the prevalence of carotid plaque by demographic characteristics and risk factors

The prevalence of carotid plaque was 41.5% overall (50.1% in men and 35.5% in women; P < 0.001). There was a significantly greater prevalence of carotid plaque in men than in women across risk factor groups. Moreover, the prevalence of carotid plaque was significantly higher in men than in women for every age and education group (all P < 0.05; Table 3).

### Sex differences in risk factors for carotid plaque

Table 4 indicates that age, education, hypertension, diabetes mellitus, and high LDL-C concentrations were associated with the risk of carotid plaque in both men and women in the univariate analysis. Moreover, alcohol consumption was a significant risk factor for carotid plaque in men.

The multivariate analysis indicated that age, hypertension, diabetes, and high LDL-C concentrations were significantly associated with the risk of carotid plaque in both men and women. The risk of carotid plaque increased with age; the corresponding adjusted odds ratios (ORs) (95% confidence intervals [CIs]) in men were 2.55 (1.94–3.37) in the 55–64-year age group, 3.64 (2.59–5.10) in the 65–74-year age group, and 9.49 (5.83–15.44) in the ≥75-year age group; the reference was the 45–54-year age group. Moreover, individuals with hypertension, diabetes mellitus, and high LDL-C concentrations had a higher risk of carotid plaque, with adjusted ORs (95% CIs) of 1.59 (1.25–2.03) for hypertension, 1.38 (1.01–1.89) for diabetes mellitus, and 4.07 (2.30–7.22) for high LDL-C concentration in men. The corresponding ORs (95% CIs) in women were 2.62 (2.04–3.36) in the 55–64-year age group, 2.95 (2.17–4.06) in the 65–74-year age group, and 5.53 (3.61–8.47) in the ≥75-year age group; in women, adjusted ORs (95% CIs) were 1.45 (1.17–1.79) for hypertension, 1.57 (1.21–2.03) for diabetes mellitus, and 5.11 (3.39–7.70) for high LDL-C concentration (Table 5).

## Discussion

This is the first population-based report of the prevalence rates of carotid plaque and relevant risk factors in a low-income population in China. We reported a carotid plaque prevalence of 41.5% overall, with a significantly higher prevalence in men (50.1%) than in women (35.5%). Age, hypertension, diabetes mellitus, and high LDL-C concentration were risk factors for carotid plaque in both men and women.

There is currently great controversy regarding the prevalence of carotid plaque, because the reported prevalence varies widely, ranging from 13.3% to 78%^15^,^23^,^24^,^25^. The Northern Manhattan Cohort Study, a population-based cohort study with a unique race/ethnicity profile, reported an overall carotid plaque prevalence of 57% among community-dwelling residents aged ≥39 years^26^. Similar prevalence rates were reported in other studies, with rates ranging from 47–58%^15^,^26^,^27^,^28^,^29^. The San Daniele Project, a population-based asymptomatic carotid atherosclerosis study in Italy, reported a lower prevalence rate of carotid plaque (13.3% in men and 13.4% in women) in 1992^30^. The highest reported prevalence rate of carotid plaque was in Americans aged 55–80 years among Humana Health System residents in 2012; carotid plaque was identified in 78% of these individuals^25^. Recently, several studies have reported the prevalence of carotid plaque in Chinese populations. A lower prevalence has been reported in Hong Kong and Taiwan (14.5% and 14.3%, respectively)^31^,^32^. The prevalence of carotid plaque was reported to be 52% for working Chinese subjects aged 40 years and older^24^,^25^ and 60.3% among urban residents aged 43–81 years in Beijing^33^. In contrast to these two studies, we observed a lower prevalence of carotid plaque in a low-income rural population, with a rate of 41.5% overall. Ethnic diversity or socioeconomic status could partly explain these differences, although the mechanisms involved remain to be established^26^,^34^. Furthermore, variations in the definitions of carotid plaque, study designs, and age structures of the study populations may have contributed to the differences in the reported prevalence rates of carotid plaque. In addition, consistent with previous studies, we observed a higher prevalence in men than in women^35^,^36^. Estrogens play a fundamental role in most aspects of plaque growth, conferring a cardiovascular advantage in women^37^. Moreover, atherosclerosis in men exhibits inflammatory and histological features distinct from atherosclerosis features in women^38^,^39^. In addition, relative to the external carotid artery and to the common carotid artery, the internal carotid artery was larger in women than in men, and in relation to the inflow area, women have a larger outflow area^40^. Bifurcation anatomy has been implicated in the development of plaque, and sex differences in bifurcation anatomy could partly account for the sex differences in the prevalence of carotid plaque.

Although extensive research has been conducted on the associations between atherosclerotic plaque and conventional risk factors, including hypertension, diabetes mellitus, dyslipidemia, and current smoking, the relationships between conventional risk factors and carotid plaque remain controversial.

Age is considered an important risk factor for atherosclerotic plaque, and a positive relationship between the prevalence of carotid plaque and age has been reported previously. An increased prevalence of carotid plaque with increasing age has been identified in previous studies^33^,^41^,^42^,^43^,^44^. In China, the reported prevalence of carotid plaque was almost 70% in those aged ≥60 years and 80% in those aged ≥70 years^33^. In Germany, the reported prevalence of extracranial carotid artery stenosis (>50%) was approximately 6.9% in patients aged >65 years, but it increased further with age^42^,^43^. In the present study, the prevalence of carotid plaque increased with age, and there was a greater prevalence in men than in women for each age group. The reference group was individuals aged 45–54 years, and the risk of carotid plaque in men increased by 1.55-fold for those aged 55–64 years, 2.64-fold for those aged 65–74 years, and 8.49-fold for those aged 75 years and older. Similar trends were observed in women; the risk of carotid plaque increased by 1.62-fold for those aged 55–64 years, 1.95-fold for those aged 65–74 years, and 4.53-fold for those aged 75 years and older. There was a greater risk of carotid plaque in men than in women among individuals over 75 years old.

In an earlier cross-sectional investigation, low socioeconomic status was associated with an increased prevalence of atherosclerotic changes in the carotid arteries, and women with low educational levels had an increased risk of carotid artery stenosis compared to those with higher educational levels^45^. Individuals with less education were reported to have larger carotid plaques, even after adjusting for vascular risk factors^46^,^47^. Moreover, low socioeconomic status was associated with progression of atherosclerosis in a middle-aged population with subclinical atherosclerosis^34^. In this study, we noted that individuals (both men and women) with low educational levels had a greater risk of carotid plaque than did those with higher educational levels; there was an especially high prevalence of carotid plaque in illiterate women. However, the association between education level and the prevalence of carotid plaque was not significant after adjusting for other risk factors.

Traditional risk factors, including age, sex, smoking status, systolic blood pressure, diastolic blood pressure, diabetes mellitus, HDL-C concentration, and LDL-C concentration, have been reported to be associated with carotid plaque^12^,^15^,^31^,^44^,^48^,^49^,^50^. However, these risk factors could explain approximately 50% of the effects on the prevalence of carotid plaque in prior studies^15^,^49^,^50^. Our findings suggested that age, hypertension, diabetes, and LDL-C concentration were significantly associated with carotid plaque in both men and women.

There were some limitations in this study. First, the study population was from a local town in Tianjin, China, so these results may not be representative of the general Chinese population. Second, the cross-sectional design may have led to a selection bias, especially among the healthy elderly. However, excluding subjects with histories of stroke and CVD likely overcame this limitation. Moreover, all participants with carotid plaque were asymptomatic, which may have decreased bias.

## Conclusions

This study was the first to report sex differences in the prevalence of carotid plaque and associated risk factors among adults aged 45 years and older in rural China. In this cross-sectional study, we assessed these sex differences among participants aged 45 years and over. There was a conspicuously higher prevalence of carotid plaque in men than in women across age groups, educational levels, and across all risk factors. Age, hypertension, diabetes mellitus, and LDL-C concentration were significantly associated with carotid plaque in both men and women. Therefore, it is vital for physicians to be aware that conventional risk factors and other related factors are of equal importance in rural residents in China; patients should be treated accordingly in order to reduce the burden of stroke and cardiovascular disease.

## Materials and Methods

### Participants and study design

This was a population-based cross-sectional study conducted from April 2014 to January 2015. The participants were from the Tianjin Brain Study which was described previously^22^,^27^,^30^,^31^,^32^. In brief, the total population included 14,251 individuals from 18 administrative villages. Approximately 95% of the populations were low-income farmers, with a per capita disposable income of <1600 USD in 2014^51^. In 2011, the average length of education was 5.26 years. All residents aged 45 years and older without a previous history of CVD and stroke were recruited to this study, while those with a previous history of CVD and stroke were excluded.

All investigative protocols were approved by the ethics committee of Peking University First Hospital and Tianjin Medical University General Hospital; the methods were carried out in accordance with the approved guidelines, and informed consent was obtained from each participant.

### Risk factors and physical examination

This study was conducted through face-to-face interviews by trained research staff. A pre-designed questionnaire was used to collect the following information: demographic information (including name, sex, date of birth, and educational level), individual and family medical history (including the presence or occurrence of hypertension, diabetes mellitus, stroke, transient ischemia attack, and coronary heart disease), and lifestyle factors (including cigarette smoking [defined as ≥1 cigarette per day for ≥1 year], passive smoking [defined as ≥1 person smoking in the room], and alcohol consumption [defined as drinking ≥500 g of alcohol per week for 1 year]).

A physical examination was performed to obtain information on blood pressure, height, and weight; the serum levels of fasting blood glucose, TC, TG, HDL-C, and LDL-C were measured. Carotid ultrasonography and 12-lead echocardiography were also performed. Body mass index was calculated as the individual’s weight (kg) divided by the square of the individual’s height (m^2^).

### Ultrasonography measurements

One trained technician blinded to participants’ information performed all ultrasound examinations. The patients were examined while they were in the supine position using B-mode ultrasonography (Terason 3000; Burlington, MA, US) with a 5–12 MHz linear array transducer. The extracranial carotid artery trees (i.e., the common carotid artery, the bifurcation, and the internal and external carotid arteries) on both sides were screened for plaque. Images were obtained and digitally stored according to a standard protocol^28^. Both longitudinal and transverse dynamic images of each plaque were stored.

### Definitions of risk factors

Hypertension was defined as systolic blood pressure ≥140 mmHg, diastolic blood pressure ≥90 mmHg, or taking medication for hypertension. Diabetes mellitus was defined as a fasting blood glucose concentration ≥7.0 mmol/L or taking medication for diabetes. Obesity was defined as a body mass index of ≥28.0 kg/m^2^, and overweight was defined as a body mass index of 24.0–27.9 kg/m^2^ 52. High TC was defined as a TC concentration of ≥6.22 mmol/L. High TG was defined as a triglyceride value of ≥2.26 mmol/L. High LDL-C was defined as an LDL-C concentration of ≥4.14 mmol/L, and low HDL-C was defined as an HDL-C concentration of ≤1.04 mmol/L^26^.

Carotid plaque was defined as a focal structure encroaching into the arterial lumen by at least 0.5 mm or 50% of the surrounding intima-media thickness value, or demonstrated as a thickness of >1.5 mm from the intima-lumen interface to the media adventitia interface^29^. Subjects with carotid plaque were defined as those having at least one lesion overall, regardless of the actual number of carotid plaques.

### Statistical analyses

Continuous variables were presented as means and standard deviations, and sex differences were compared using Student t-tests. Categorical variables were presented as numbers with frequencies, and sex differences were compared using chi-squared tests. All participants were categorized into four age groups (45–54, 55–64, 65–74, and ≥75 years) and three education groups (0, 1–6, and >6 years). The risk factors for carotid plaque were assessed individually for men and women using logistic regression analyses. Results of the univariate analysis were presented as unadjusted ORs and 95% CIs; results of the multivariate analysis were presented as adjusted ORs and 95% CIs after adjustment for covariates. Of these, age group and education group were analyzed as categorical variables, referenced as 45–54 years and >6 years, respectively; hypertension, diabetes mellitus, obesity, current smoking, passive smoking, alcohol consumption, and high TC, high TG, low HDL-C, and high LDL-C levels were analyzed as dichotomous variables. P values < 0.05, <0.0166, and <0.0125 were considered statistically significant for dichotomous variables, categorical variables with three groups, and categorical variables with four groups, respectively. SPSS for Windows (version 15.0; SPSS Inc., Chicago, IL, USA) was used for analyses.

## Additional Information

**How to cite this article**: Zhao, W. *et al*. Sex Differences in Prevalence of and Risk Factors for Carotid Plaque among Adults: A Population-based Cross-Sectional Study in Rural China. *Sci. Rep.* **6**, 38618; doi: 10.1038/srep38618 (2016).

**Publisher's note:** Springer Nature remains neutral with regard to jurisdictional claims in published maps and institutional affiliations.

## Figures and Tables

**Table 1:** Sex differences in demographic characteristics and prevalence of cardiovascular disease risk factor in the participants.

Characteristics	Men	Women	P	Cohen’s D
Total, n (%)	1560 (41.2)	2229 (58.8)		—
Age, years, mean (SD)	61.13 (9.90)	59.07 (9.47)	<0.001	0.27
Age group, n (%)				0.22
45~54 years	430 (27.6)	806 (36.2)	<0.001	
55~64 years	632 (40.5)	882 (39.6)	0.560	
65~74 years	338 (21.7)	386 (17.3)	0.001	
≥75 years	160 (10.3)	155 (7.0)	<0.001	
Education level, year, mean (SD)	6.40 (3.22)	4.84 (3.61)	<0.001	0.45
Education group, n (%)				0.54
0 years	137 (8.8)	522 (23.4)	<0.001	
1~6 years	699 (44.8)	995 (44.6)	0.947	
>6 years	724 (46.4)	712 (31.9)	<0.001	
SBP, mmHg, mean (SD)	147.76 (21.41)	145.49 (22.64)	0.002	0.10
DBP, mmHg, mean (SD)	88.50 (11.22)	85.62 (11.39)	<0.001	0.25
BMI, Kg/m^2^, mean (SD)	25.20 (3.44)	25.82 (3.82)	<0.001	0.17
FG, mmol/l, mean (SD)	5.91 (1.42)	5.93 (1.67)	0.660	0.01
TC, mmol/L, mean (SD)	4.62 (1.00)	5.04 (1.11)	<0.001	0.40
TG, mmol/L, mean (SD)	1.61 (1.24)	1.87 (1.22)	<0.001	0.21
HDL-C, mmol/L, mean (SD)	1.39 (0.43)	1.50 (0.48)	<0.001	0.24
LDL-C, mmol/L, mean (SD)	2.61 (1.20)	2.76 (1.28)	<0.001	0.12

**Table 2:** Sex differences in the prevalence of cardiovascular disease risk factors in the participants.

Characteristics	Men	Women	P	Cohen’s D
Hypertension	1111 (71.2)	1472 (66.0)	0.001	0.11
Diabetes	216 (14.1)	317 (14.5)	0.719	0.01
Obesity	323 (20.7)	565 (25.3)	0.001	0.11
Current smoking	896 (57.4)	53 (2.4)	<0.001	1.11
Passive smoking	246 (15.8)	876 (39.3)	<0.001	0.64
Alcohol consumption	561 (36.0)	30 (1.3)	<0.001	0.72
High TC	99 (6.4)	290 (13.2)	<0.001	0.28
High TG	273 (17.8)	543 (24.8)	<0.001	0.18
Low HDL-C	294 (19.1)	252 (11.5)	<0.001	0.19
High LDL-C	101 (6.6)	195 (8.9)	0.010	0.09

**Table 3:** Sex differences in the prevalence of carotid plaque in the participants by demographical characteristics and risk factors in all participant.

Characteristics	Men	Women	P
Total	782 (50.1)	792 (35.5)	<0.0001
Age group			
45~54 yrs	125 (29.1)	156 (19.4)	<0.0001
55~64 yrs	323 (51.1)	361 (40.9)	<0.0001
65~74 yrs	208 (61.5)	182 (47.2)	<0.0001
≥75 years	126 (78.8)	93 (60.0)	<0.0001
Education level			
0 year	79 (57.7)	239 (45.8)	0.013
1~6 years	390 (55.8)	369 (37.1)	<0.0001
>6 years	313 (43.2)	184 (25.8)	<0.0001
Hypertension	610 (54.9)	603 (41.0)	<0.0001
NO hypertension	172 (38.3)	189 (25.0)	<0.0001
Diabetes	125 (57.9)	157 (49.5)	<0.058
No diabetes	641 (48.6)	622 (33.2)	<0.0001
Obesity	149 (46.1)	202 (35.8)	0.002
NO obesity	633 (51.2)	590 (35.5)	<0.0001
Smoking	445 (49.7)	24 (45.3)	0.535
Never smoking	337 (50.8)	768 (35.3)	<0.0001
Passive smoking	111 (45.1)	291 (33.2)	0.001
NO passive smoking	671 (51.1)	501 (37.0)	<0.0001
Alcohol consumption	250 (44.6)	13 (43.3)	0.895
NO alcohol consumption	532 (53.3)	779 (35.4)	<0.0001
High TC	61 (61.6)	135 (46.6)	0.010
Normal TC	705 (49.1)	644 (33.9)	<0.0001
High TG	127 (46.5)	202 (37.2)	0,010
Normal TG	639 (50.6)	577 (35.1)	<0.0001
Low HDL-C	145 (49.3)	89 (35.3)	0.001
Norma HDL-C	621 (50.0)	690 (35.6)	<0.0001
High LDL-C	76 (75.2)	131 (67.2)	0.151
NormaI LDL-C	690 (48.1)	648 (32.5)	<0.0001

*Indicated all data was presented as n (%).

**Table 4:** Sex differences in the prevalence of carotid plaque in the participants by demographical characteristics and risk factors in all participant^*^.

Characteristics	Men		Women	
	Rate (%)	P	Rate (%)	P
Total	782 (50.1)		792 (35.5)	<0.001
Age group		<0.001		<0.001
45~54 yrs	125 (29.1)		156 (19.4)	
55~64 yrs	323 (51.1)		361 (40.9)	
65~74 yrs	208 (61.5)		182 (47.2)	
≥75 years	126 (78.8)		93 (60.0)	
Education level:		<0.001		<0.001
0 year	79 (57.7)		239 (45.8)	
1~6 years	390 (55.8)		369 (37.1)	
>6 years	313 (43.2)		184 (25.8)	
Hypertension		<0.001		<0.001
Yes	172 (38.3)		189 (25.0)	
No	610 (54.9)		603 (41.0)	
Diabetes		0.011		<0.001
Yes	641 (48.6)		622 (33.2)	
No	125 (57.9)		157 (49.5)	
Obesity		0.107		0.899
Yes	633 (51.2)		590 (35.5)	
No	149 (46.1)		202 (35.8)	
Smoking		0.671		0.133
Yes	337 (50.8)		768 (35.3)	
No	445 (49.7)		24 (45.3)	
Passive smoking		0.087		0.066
Yes	671 (51.1)		501 (37.0)	
No	111 (45.1)		291 (33.2)	
Alcohol consumption		0.001		0.369
Yes	532 (53.3)		779 (35.4)	
No	250 (44.6)		13 (43.3)	
High TC		0.016		<0.001
Yes	705 (49.1)		644 (33.9)	
No	61 (61.6)		135 (46.6)	
High TG		0.222		0.365
Yes	639 (50.6)		577 (35.1)	
No	127 (46.5)		202 (37.2)	
Low HDL-C		0.834		0.924
Yes	621 (50.0)		690 (35.6)	
No	145 (49.3)		89 (35.3)	
High LDL-C		<0.001		<0.001
Yes	690 (48.1)		648 (32.5)	
No	76 (75.2)		131 (67.2)	

**Table 5:** The unadjusted and adjusted OR with 95% CI for risk factors of carotid plaque by gender in univariate and multivariate analysis.

Characteristics	Reference	Unadjusted OR (95% CI)		Adjusted OR (95% CI)	
		Men	Women	Men	Women
Age group	45–54yrs	1.00	1.00	1.00	1.00
55~64 years		2.55 (1.97, 3.31)^*^	2.89 (2.32, 3.60)^*^	2.55 (1.94, 3.37)^*^	2.62 (2.04, 3.36)^*^
65~74 years		3.90 (2.89, 5.28)^*^	3.72 (2.85, 4.85)^*^	3.64 (2.59, 5.10)^*^	2.95 (2.17, 4.06)^*^
≥75 yrs		9.04 (5.89, 10.20)^*^	6.25 (4.34, 9.01)^*^	9.49 (5.83, 15.44)^*^	5.53. (3.61, 8.47)^*^
Education level	>6yrs	1.00	1.00	1.00	1.00
0 year		1.79 (1.24, 2.59)^*^	2.42 (1.91, 3.08)^*^	0.71 (0.45, 1.09)	1.07 (0.80, 1.44)
1~6 yrs		1.66 (1.34, 2.04)^*^	1.69 (1.37, 2.09)^*^	1.08 (0.85, 1.38)	1.03 (0.80, 1.31)
Hypertension	No	1.96 (1.57, 2.45)^*^	2.09 (1.72, 2.53)^*^	1.59 (1.25,2.03)^*^	1.45 (1.17, 1.79)^*^
Diabetes	No	1.46 (1.09, 1.95)^*^	1.97 (1.55, 2.51)^*^	1.38 (1.01,1.89)^*^	1.57 (1.21, 2.03)^*^
Obesity	No	0.82 (0.64, 1.04)	1.01 (0.83, 1.24)	—	—
Current smoking	No	0.96 (0.78, 1.17)	1.52 (0.88, 2.62)	—	—
Passive smoking	No	0.79 (0.60, 1.04)	0.85 (0.71, 1.01)	—	—
Alcohol consumption	No	0.71 (0.57, 0.87)^*^	1.39 (0.67, 2.89)	0.81 (0.64,1.01)	—
High TC	No	1.67 (1.10, 2.53)^*^	1.70 (1.32, 2.18)^*^	0.84 (0.49,1.45)	0.66 (0.47, 2.94)
High TG	No	0.85 (0.65, 1.10)	1.10 (0.90, 1.34)	—	—
Low HDL-C	No	0.97 (0.76, 1.26)	0.99 (0.75, 1.30)	—	—
High LDL-C	No	3.28 (2.07, 5.22)^*^	4.25 (3.11, 5.82)^*^	4.07 (2.30,7.22)^*^	5.11 (3.39, 7.70)^*^

*Indicated P < 0.05 in univariate/multivariable analysis.
